# Intralesional Platelet-Rich Plasma for Treating Chronic Peyronie’s Disease: A Single-Center Retrospective Cohort Study

**DOI:** 10.3390/medicina62010221

**Published:** 2026-01-21

**Authors:** Luigi Pucci, Celeste Manfredi, Catello Sansone, Simone Tammaro, Giorgio Stanziola, Nunzio Langella, Giuseppe Dachille, Davide Arcaniolo, Marco De Sio, Maurizio Carrino

**Affiliations:** 1 Department of Urology, AORN Antonio Cardarelli, 80131 Naples, Italy; luigi.pucci@hotmail.it (L.P.); giorgio.stanziola@aocardarelli.it (G.S.); nunzio.langella@aocardarelli.it (N.L.); maurizio.carrino@aocardarelli.it (M.C.); 2 Urology Unit, Department of Woman, Child and General and Specialized Surgery, University of Campania “Luigi Vanvitelli”, 80131 Naples, Italy; manfredi.celeste@gmail.com (C.M.); davide.arcaniolo@gmail.com (D.A.); marco.desio@unicampania.it (M.D.S.); 3 Department of Neurosciences, Reproductive Sciences and Odontostomatology, University of Naples “Federico II”, 80138 Napoli, Italy; catellosansone@gmail.com; 4 Andrology and Kidney Transplantation Unit, Department of Precision and Regenerative Medicine and Ionian Area Urology, University of Bari, 70121 Bari, Italy; gdachille@hotmail.com

**Keywords:** platelet-rich plasma, Peyronie’s disease, penile curvature, regenerative therapy

## Abstract

*Background and Objectives*: Peyronie’s disease (PD) is a chronic fibrotic disorder of the tunica albuginea causing penile deformity and sexual dysfunction. Platelet-rich plasma (PRP) has been proposed as a regenerative therapy with potential disease-modifying properties, but evidence of its use in chronic PD is scarce. This study evaluated the efficacy and safety of intralesional PRP injections in men with stable PD. *Materials and Methods*: A single-center retrospective cohort study was conducted including men with chronic PD treated with three weekly intralesional PRP injections (April 2022–April 2025). Inclusion required curvature stability for ≥6 months and absence of prior PD therapy. The primary outcome was a change in penile curvature at 4 weeks post-treatment. Secondary outcomes included plaque thickness (ultrasound), erectile function (IIEF-5), and safety (Clavien–Dindo grading). *Results*: Thirty-six men (mean age 61.2 ± 10.4 years) completed the treatment. Mean penile curvature decreased from 30.5 ± 7.3° to 24.2 ± 8.3° (Δ = −6.3°, 95% CI −7.7 to −5.3; *p* < 0.001); 25% achieved a ≥10° reduction. Mean plaque thickness declined from 3.25 ± 0.69 mm to 2.91 ± 0.76 mm (Δ = −0.34 mm; *p* < 0.001). IIEF-5 increased modestly (+1.1; *p* = 0.142). Only mild, transient adverse events occurred (pain 5.6%, hematoma 2.8%). *Conclusions*: Intralesional PRP was safe and yielded statistically significant but modest reductions in penile curvature and plaque thickness in chronic PD. Clinically meaningful improvement occurred in a minority of patients. These findings support keeping PRP investigational pending well-designed randomized controlled trials with standardized protocols and longer follow-up.

## 1. Introduction

Peyronie’s disease (PD) is a fibrotic disorder of the tunica albuginea characterized by inelastic plaque formation and penile deformity. Its pathophysiology involves microvascular trauma, aberrant wound healing, and dysregulated fibroblast activity, leading to excess collagen deposition and loss of tunical elasticity [1].

Clinically, the burden of PD extends beyond deformity magnitude and includes sexual dysfunction, reduced sexual confidence, and partner-related intimacy concerns. Consequently, this condition is often accompanied by significant psychological distress and has an impact on the patient’s quality of life. This is particularly relevant when counseling patients in real-world settings, where treatment decisions are driven by functional impact and patient needs as much as by objective measurements. Accordingly, incorporating patient-centered considerations is essential when interpreting outcomes and when designing studies aimed at clinically meaningful endpoints [1,2].

Therapeutic strategies in PD are generally tailored to the disease stage. Traditionally, an acute (active) phase—typically lasting up to 12–18 months—has been distinguished, marked by evolving deformity and often, but not invariably, penile pain, from a chronic (stable) phase characterized by curvature stability and resolution of pain. However, the definition of these phases is not standardized and varies among studies, and the transition between them is not sharply demarcated. Management approaches differ accordingly: conservative options are usually favored in the acute phase, aiming to limit curvature progression and alleviate pain, whereas surgery is considered the gold standard in the chronic phase for correcting functionally limiting deformities [1,2,3].

In clinical practice, however, a substantial proportion of men in the stable phase either postpone or decline surgery, even when the deformity is functionally significant, because of personal preference, perceived invasiveness, fear of penile shortening, concerns about postoperative sexual function, or limited access to specialized surgical pathways. This results in a frequent clinical scenario in which men with stable disease seek conservative treatments primarily aimed at reducing functional impairment and psychological burden. Beyond representing a potential alternative to surgery in selected stable cases, conservative therapy, by improving penile deformity, may also reduce the complexity and the risk of complications associated with any subsequent surgical intervention [1,2].

Among conservative options, intralesional therapy is a key modality. In addition to established agents such as collagenase Clostridium histolyticum (CCH), other injectables remain under investigation, including hyaluronic acid and platelet-rich plasma (PRP). PRP belongs to the broader category of regenerative therapies, which have attracted increasing attention for their potential disease-modifying effects. PRP is an autologous concentrate of activated platelets suspended in plasma that delivers high concentrations of growth factors and cytokines capable of modulating inflammation and influencing extracellular matrix remodeling [2]. From a biological standpoint, the rationale for PRP in PD stems from its potential to influence profibrotic signaling and extracellular matrix turnover, processes central to tunical fibrosis and plaque maturation.

Preliminary clinical reports suggest that PRP may reduce curvature and plaque size and improve PD-related symptoms, including penile pain and erectile function, with generally mild and transient adverse events [4,5,6,7]. Despite these promising signals, the current evidence base remains limited. A 2024 systematic review identified only a handful of PD studies, with marked heterogeneity in PRP preparation protocols, dosing regimens, and concomitant therapies, along with small sample sizes, short follow-up, and important methodological constraints [2,3]. Consequently, the current European Association of Urology (EAU) guidelines still classify PRP as experimental and recommend its use be restricted to clinical trials [8].

Evidence on PRP focusing exclusively on chronic PD is particularly scarce, largely limited to small prospective cohorts and pilot studies that often lack adequate methodologies [4,5,6,7]. This leaves a significant knowledge gap for a clinically relevant subgroup: men with stable disease and functionally limiting deformities who are unwilling to undergo surgery. Bridging this gap is essential to deliver informed counseling, tailor therapeutic strategies to this neglected patient subgroup, and establish the evidence base required to support future high-quality randomized controlled trials.

This study aimed to evaluate the efficacy and safety of intralesional PRP injections in men with chronic PD, critically interpreting the findings in the context of prior PRP studies in this setting.

## 2. Materials and Methods

### 2.1. Study Design and Ethical Details

We conducted a single-center retrospective cohort study including consecutive men treated with intralesional PRP for chronic PD between April 2021 and December 2024 at Antonio Cardarelli Hospital (Napoli, Italy).

Data were prospectively collected using a predefined standardized template and subsequently extracted from electronic medical records for the purposes of the present retrospective analysis. The collected dataset covered demographics, comorbidities, disease characteristics, procedural details, and outcomes. Baseline and follow-up assessments were those routinely performed in clinical practice and recorded contemporaneously at the time of each visit. All data were pseudonymized prior to analysis in accordance with applicable data protection regulations. Complete-case analyses were prespecified given the retrospective nature of the study; patients with missing key outcome measures required for within-patient comparisons were excluded, as stated above. The study was reported in line with transparent reporting principles for observational research.

The study complied with institutional policies and the Declaration of Helsinki [9] and was approved by Ethics Committee (protocol NO. 0012112/11/02/2021). All patients provided written informed consent for the off-label procedure and for the use of anonymized data for research purposes.

### 2.2. Eligibility Criteria

The inclusion criteria were as follows: adult men; chronic PD (defined as absence of penile pain and no patient-reported worsening of deformity over the preceding 6 months, following a prior active phase during which symptoms and/or plaque first appeared and progressed); functionally significant deformity interfering with sexual intercourse (patient-reported); dorsal, lateral, or dorsolateral curvature; tunical plaque detectable on palpation; no prior therapy for PD; not seeking surgery.

The exclusion criteria were as follows: calcified plaques on ultrasound; penile curvature ≥ 60°; ventral curvature; complex deformities (hinge or hourglass); men who reported no partnered penetrative sexual activity or solitary sexual activity only; penile prosthesis; history of priapism; history or suspicion of penile tumor; hematologic disorders; antiplatelet or anticoagulant therapy (except acetylsalicylic acid 100 mg); suspected cutaneous infection at the injection site; significant immunosuppression; poorly controlled diabetes mellitus; untreated or unstable hypogonadism; psychiatric disorders. Any other condition judged by the treating physician to be unsafe for PRP injection or that prevented penetrative intercourse was exclusionary. Patients with missing data were excluded. Use of phosphodiesterase type-5 inhibitors (PDE5Is) was permitted provided the regimen (agent, dose, schedule) had been stable for ≥4 weeks before baseline and remained unchanged throughout treatment period and follow-up. The use of other erectile dysfunction (ED) treatments during the study period was prohibited. Patients who had received intracavernosal vasoactive injections, low-intensity extracorporeal shockwave therapy (Li-ESWT), or vacuum device treatment for ED within the previous 12 months were excluded.

### 2.3. PRP Preparation and Injection Protocol

PRP was prepared from a single autologous peripheral venous blood draw using a closed, sterile kit (IMP.A.C.T.^®^ APC, IMPACT International GmbH, Cologne, Germany), according to the manufacturer instructions. Whole blood was centrifuged at 2800 rpm for 5 min to obtain PRP. Because the study reflects a real-world retrospective workflow, quantitative characterization of the final PRP product (for example, platelet concentration, leukocyte content, or activation status) was not routinely available and therefore could not be reported. The index plaque was established by palpation and confirmed/targeted with ultrasound. It was defined as the palpable tunical plaque considered the predominant contributor to deformity (typically located at or near the point of maximal curvature). When multiple plaques were present, the largest meeting these criteria was designated the index plaque. The overlying skin was marked with a dermatographic pen.

After local anesthesia (dorsal penile nerve block with 1% lidocaine 10 mL) and skin antisepsis, 12 mL of PRP was infiltrated intralesionally and perilesionally using a 21-gauge needle on a detumescent penis. Patients received one session per week for three consecutive weeks. All injections were performed by the same experienced andrologist. No antibiotic prophylaxis or analgesics were prescribed. No manual or vacuum-assisted penile modeling was performed as adjunctive treatment. Patients were advised to abstain from sexual activity for 24 h after the injection.

### 2.4. Outcomes and Patient Assessments

Data abstracted from records included demographics and baseline clinical characteristics. Efficacy assessments were performed in person at baseline and 4 weeks after completion of the 3-injection course and included penile curvature (primary outcome) according to the Kelami method [10]; index plaque thickness on penile ultrasound; and erectile function using the 5-item version of the International Index of Erectile Function (IIEF-5) [11].

Safety outcomes included intra-procedural complications and post-procedural adverse events, the latter graded according to the Clavien–Dindo (CD) classification and recorded during the treatment period and at the final visit (4 weeks after completion of the injection cycle) [12].

All patient evaluations were performed by experienced andrologists. Penile ultrasound examinations, including plaque thickness measurements, were performed by the same operator at baseline and follow-up using a standardized protocol using a high-frequency linear transducer. Given the observational design, assessor blinding was not feasible. Plaque thickness was recorded in millimeters using electronic calipers on B-mode imaging at the point of maximal thickness of the index plaque, applying the same operational definition at baseline and follow-up to maximize within-patient comparability. Penile curvature was assessed using a medical goniometer on standardized self-photographs of the erect penis obtained at the time of maximal spontaneous erection. Patients were instructed to acquire standardized images in two orthogonal views under maximal rigidity. Curvature was then measured on these images using a medical goniometer during the in-person clinical assessment.

### 2.5. Statistical Analysis

Given the retrospective design, no prospective sample size calculation was performed. Before data retrieval, we considered a 10° reduction in penile curvature as clinically meaningful, consistent with prior studies [5,6,7]. Under conservative assumptions for the Standard Deviation (SD) of the paired change (SDΔ = 15°) [13], a two-sided α = 0.05 and 80% power would require ≥18 patients to detect Δ = 10°.

The distribution of paired differences was assessed using the Shapiro–Wilk test [14]. Continuous variables were reported as mean ± SD or median (interquartile range [IQR]), as appropriate, and categorical variables as counts and percentages. All analyses were within-patient (pre- vs. post-treatment). Continuous outcomes (curvature, plaque thickness, IIEF-5) were compared using a paired *t*-test or Wilcoxon signed-rank test according to data normality [15]. The results were reported as mean paired changes with two-sided 95% confidence intervals (Cis) based on the observed SDΔ, with two-sided *p*-values provided as complementary information, and were graphically displayed as paired estimation plots (Gardner–Altman style) [16]. In addition, a responder analysis was performed, reporting the proportion of patients achieving a ≥10° curvature reduction, with 95% binomial CIs. No imputation was performed (complete-case analyses). Two-sided *p* < 0.05 was considered statistically significant. Analyses were conducted in RStudio v.2024.09.0+375 (Posit, Boston, MA, USA).

## 3. Results

Thirty-six men were included. The mean age was 61.2 ± 10.4 years, and the mean disease duration was 19.2 ± 5.8 months. At baseline, the mean penile curvature was 30.5 ± 7.26°. Most patients had a single palpable plaque (28/36, 77.8%) (Table 1).

**Figure 1:**
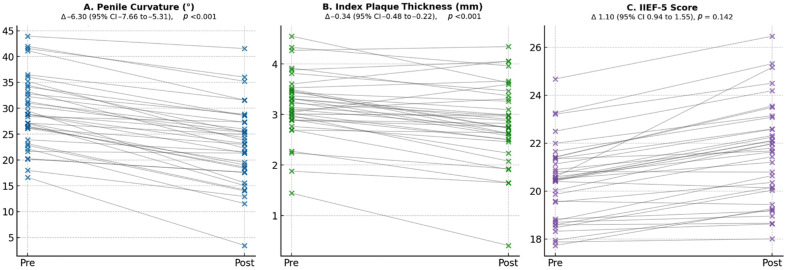
PRP efficacy outcomes: penile curvature, plaque thickness, and IIEF-5. CI: Confidence Interval; IIEF-5: 5-item version of the International Index of Erectile Function; PD: Peyronie’s disease; PRP: platelet-rich plasma. Paired estimation plots showing pre-treatment (Pre) and post-treatment (Post; 4 weeks after completion of the PRP treatment cycle) values for (**A**) penile curvature (degrees), (**B**) index plaque thickness (mm), and (**C**) IIEF-5 score in 36 patients with PD. Each gray line represents an individual patient’s change from baseline to follow-up. Mean paired changes (Δ) with 95% CI and two-sided *p*-values from paired *t*-tests are reported above each plot.

All patients completed the full injection protocol and were reassessed 4 weeks after the last injection. Mean penile curvature decreased from 30.5 ± 7.26° pre-treatment to 24.2 ± 8.30° post-treatment (mean change −6.3°; 95% Cl −7.66 to −5.31; *p* < 0.001). Nine patients (25%; 95% CI 12.1–42.2%) achieved a ≥10° curvature reduction. The mean index plaque thickness declined from 3.25 ± 0.69 mm to 2.91 ± 0.76 mm (mean change −0.34 mm; *p* < 0.001). The IIEF-5 score increased from 20.0 ± 1.72 to 21.1 ± 1.89 (mean change +1.1 points; *p* = 0.142) (Figure 1). Treatment was well tolerated. No intraprocedural complications occurred. Post-procedurally, only mild injection-site pain (CD grade I) in two (5.6%) patients and a superficial hematoma (CD grade I) in one (2.8%) subject were recorded. No major adverse events (CD ≥ grade III) were observed.

## 4. Discussion

### 4.1. Main Findings and Previous Literature

In this single-center cohort of men with chronic PD, three weekly intralesional PRP injections produced a modest reduction in penile curvature at 4 weeks (mean change −6.3°), a small reduction in ultrasound plaque thickness (−0.34 mm), and no statistically significant change in IIEF-5. Cross-study comparisons are possible but should be interpreted cautiously, given the substantial heterogeneity in PRP preparation, dosing, injection protocols, assessment tools, follow-up windows, and—crucially—patient/disease characteristics and how “acute” versus “chronic” phases are defined across studies. In addition, PRP can be combined with other modalities (e.g., Li-ESWT, penile modeling, vacuum device), making it difficult to isolate its independent effect [2].

In our cohort, although the overall improvement in curvature was statistically significant, the mean change was 6.3°, and the ~10° threshold commonly regarded as clinically relevant in non-surgical PD studies [4,5,6] was achieved in only 25% of cases. In addition, our curvature effect sits on the lower end of published PRP experiences. In chronic-phase monotherapy studies, Achraf et al. (n = 65; ~6 injections) reported mean curvature reductions of −16.9° and −17.3° across two baseline-severity strata [5], while Dachille et al. (n = 72; three injections) observed a median decrease from 50° to 40° by ~6 weeks [6]—larger than our change, likely reflecting higher baseline curvature and protocol differences. Chronic-phase combination protocols generally yield larger angle changes compared to ours: Ergün & Sağır (n = 26; PRP + Li-ESWT) observed an average improvement of ~10° [7]; Alshuaibi et al. (n = 36; PRP + PNT + penile modeling) reported a mean reduction of −16.85° ± 14.81° [12]; and Zugail et al. (n = 54; PRP + PNT + vacuum device) documented a decrease from 45° to 30° (−14.1°) [17]—effects that cannot be attributed solely to PRP. Mixed-phase cohorts provide additional context. In the randomized, placebo-controlled, crossover trial by Ledesma et al. (n = 41 enrolled; 28 analyzed; monotherapy), curvature improvement reached statistical significance only in the PRP→placebo sequence at 6 months (median 40° → 25°, −15°) [18], suggesting that our 4-week post-course assessment may underestimate eventual effects. Similarly, Virag et al. (n = 90; PRP + hyaluronic acid) reported clinically meaningful curvature reductions over the 2-month protocol, although the additive role of hyaluronic acid remains uncertain [19]. Our smaller effect likely reflects a combination of factors, most notably the relatively low baseline curvature in our cohort (mean ~30°), which imposes an intrinsic ceiling effect on the magnitude of achievable absolute improvement. Additional contributors include the short post-treatment assessment interval and the use of PRP as monotherapy, without adjunctive interventions beyond injections. Furthermore, small absolute curvature changes may fall within the range of measurement error, which is influenced by erection rigidity, photographic angle, and the measurement technique itself [9].

Regarding plaque size, Dachille et al. reported a median reduction from 11.1 mm to 8.2 mm (−2.9 mm) [6], whereas in our cohort, the mean change was −0.34 mm. This discrepancy may be attributable to our smaller baseline measurements-likely because we measured thickness rather than maximal length—and to the earlier reassessment. However, ultrasound plaque sizing is operator- and protocol-dependent, and the mean change we observed is within the range of technical sensitivity; consequently, our findings should be interpreted with caution. Notably, plaque-size change can dissociate from curvature outcomes; for example, in the chronic-phase combination series by Ergün and Sağır (PRP + Li-ESWT), penile curvature improved while plaque size remained statistically unchanged [7]. We observed no significant change in IIEF-5, consistent with the lack of improvement in Dachille et al. [6].

Our safety profile-only mild, transient grade I events (injection-site pain or superficial hematoma)-matches prior reports of low rates of minor adverse effects and absence of major complications with intralesional PRP [2,4,6,17].

The clinical findings observed in our cohort are supported by a coherent pathophysiological rationale. Preclinical evidence, primarily derived from in vitro models not specifically designed for PD, indicates that PRP can modulate key profibrotic pathways relevant to this condition, including TGF-β1-mediated fibroblast-to-myofibroblast differentiation and extracellular matrix production [20,21,22,23]. These mechanisms are central to tunical fibrosis, providing biological plausibility for a potential disease-modifying role of PRP in PD. Importantly, this preclinical rationale has been subsequently supported by multiple preliminary clinical studies, including prospective cohorts and a randomized placebo-controlled crossover trial, which have reported signals of efficacy together with a favorable safety profile [2,3,5,6,7]. Within this framework, our results add clinical evidence in a well-defined chronic-phase population, further supporting the translational continuum from experimental models to early clinical investigation. From a clinical standpoint, these findings are primarily relevant for counseling men with stable/chronic PD who are considering nonsurgical management. The observed heterogeneity of responses, with clinically meaningful curvature reduction confined to a minority of patients, underscores the importance of structured expectation management and highlights the limitations of relying on mean changes alone. In this context, responder-based endpoints and patient-centered outcomes capturing functional interference and distress are likely to be more clinically informative than average angle reduction. Finally, given the short post-treatment assessment window, these results should be interpreted as an early signal rather than a definitive estimate of durability, while still providing useful guidance for the design of future controlled studies in terms of eligibility criteria and realistic effect-size assumptions.

### 4.2. Strengths and Limitations

A key strength of this study is the clear definition of chronic-phase PD and the use of stringent inclusion/exclusion criteria, yielding a relatively uniform cohort. Outcome assessment relied on validated tools, enhancing measurement reliability, and cross-study comparability. Procedures were ultrasound-targeted and performed by a single experienced andrologist, reducing operational variability.

The main limitations of this study are the retrospective, uncontrolled design, entailing risks of regression to the mean, placebo, and “needling” effects and the short follow-up (4 weeks post-course), which may miss delayed responses reported in randomized data [17]. Baseline deformity was modest, constraining the absolute improvement detectable. Small absolute curvature changes can fall within the measurement error, and ultrasound plaque sizing is operator- and protocol-dependent. We did not collect PD-specific patient-reported outcomes (e.g., Peyronie’s Disease Questionnaire [PDQ], patient satisfaction), which reduces clinical sensitivity. The relatively small sample and brief observation window limit detection of rare or delayed adverse events. PRP was not quantitatively characterized (e.g., platelet concentration/activation), limiting reproducibility. Although PDE5I regimens were kept constant and other ED therapies were explicitly prohibited, adherence could not be independently verified. Finally, generalizability is restricted to men with chronic PD and mild-to-moderate curvature, in addition, our stringent selection criteria further narrow external applicability.

### 4.3. Future Perspectives

Randomized controlled trials in chronic-phase PD are needed. PRP protocols should be standardized and transparently reported (baseline platelet count and fold increase; leukocyte/RBC content; activation method; injectate volume; needle gauge and number of passes/needling; time from draw to injection). Future studies should directly compare candidate protocols to identify an optimal regimen. Sample sizes must be adequately powered, with longer follow-up to assess onset, durability, and any delayed effects. Trials should prespecify clinically meaningful primary endpoints (e.g., proportion achieving ≥10° curvature reduction) and stratify analyses by baseline curvature bands. Appropriate comparators are essential to isolate any PRP effect (e.g., sham/needling alone, saline, hyaluronic acid), with strict control of concomitant treatments (PDE5Is, vacuum device, Li-ESWT). Given the largely out-of-pocket landscape in healthcare, economic evaluations from payer and patient perspectives should be incorporated. Ultimately, such studies should generate the high-quality evidence needed to support specific guideline recommendations.

## 5. Conclusions

In men with chronic PD, three weekly intralesional PRP injections were well tolerated and yielded a statistically significant curvature reduction at 4 weeks. However, the mean change was clinically meaningful in only a minority of patients, and this finding is further tempered by concerns regarding potential measurement errors and the relatively mild baseline curvature. Taken together with mixed, heterogeneous prior literature, these findings support cautious optimism and keeping PRP investigational in chronic PD. Well-designed randomized trials are needed to clarify the magnitude, onset, and durability of PRP’s effects in this setting.

## Figures and Tables

**Table 1:** Baseline demographic and clinical characteristics of patients undergoing PRP.

Number of patients	36
Age, mean (SD), years	61.2 (10.4)
BMI, mean (SD) Kg/m^2^	24.3 (2.5)
Smoking habits, n (%)	15 (42)
Diabetes, n (%)	14 (38)
SPL, mean (SD), cm	12.08 (1.86)
Duration of PD, mean (SD), months	19.2 (5.8)
Penile curvature, mean (SD), degrees	30.5 (7.26)
Direction of curvature, n (%)	
▪ Lateral	15 (41.7)
▪ Dorsal	13 (36.1)
▪ Dorsolateral	8 (22.2)
Penile plaque, n (%)	
▪ 1	28 (77.8)
▪ 2	6 (16.7)
▪ >2	2 (5.6)
Index penile plaque thickness, mean (SD), mm	3.25 (0.69)
IIEF-5, mean (SD), points	20.0 (1.72)

IIEF-5: 5-item version of the International Index of Erectile Function; PD: Peyronie’s disease; PRP: platelet-rich plasma; SD: Standard Deviation; SPL: Stretched Penile Length.

## Data Availability

The scientific data will be made available upon reasonable request to the corresponding author.

## Abbreviations

The following abbreviations are used in this manuscript:

BMI	Body Mass Index
CC BY	Creative Commons Attribution
CCH	Collagenase Clostridium histolyticum
CD	Clavien–Dindo classification
CI	Confidence Interval
CIs	Confidence Intervals
EAU	European Association of Urology
ED	Erectile Dysfunction
IIEF-5	5-item version of the International Index of Erectile Function
IQR	Interquartile Range
Li-ESWT	Low-Intensity Extracorporeal Shockwave Therapy
MD	Medical Doctor
PD	Peyronie’s Disease
PDE5Is	Phosphodiesterase Type-5 Inhibitors
PDQ	Peyronie’s Disease Questionnaire
PNT	Penile Needling Therapy
PRP	Platelet-Rich Plasma
RBC	Red Blood Cell
RPM	Revolutions Per Minute
SD	Standard Deviation
SDΔ	Standard Deviation of the Paired Difference
SPL	Stretched Penile Length
